# Index Patient and SARS Outbreak in Hong Kong

**DOI:** 10.3201/eid1002.030645

**Published:** 2004-02

**Authors:** Raymond S.M. Wong, David S. Hui

**Affiliations:** * Prince of Wales Hospital, Chinese University of Hong Kong, Shatin, New Territories, Hong Kong Special Administrative Region, People’s Republic of China

**Keywords:** Severe acute respiratory syndrome, antiviral agents, treatment

## Abstract

During the global outbreak of severe acute respiratory syndrome (SARS) in 2003, treatment was empiric. We report the case history of the index patient in a hospital outbreak of SARS in Hong Kong. The patient recovered after conventional antimicrobial therapy. Further studies are needed to address treatment of SARS, which has high attack and death rates.

Severe acute respiratory syndrome (SARS), a new disease that is highly contagious, has caused a major impact worldwide. Treatment of this disease remains empiric. This report describes the natural history of a case of SARS in a young, previously healthy patient who received no specific therapy for infection with SARS-associated coronavirus (SARS-CoV). He was the index patient in a large hospital outbreak in Prince of Wales Hospital in Hong Kong (*1*).

## Case Report

In early March 2003, a 26-year-old man was admitted to a general medical ward of the Prince of Wales Hospital; he had been ill for 1 week with fever, chills, and rigor. He had had a cough productive of whitish sputum for 2 weeks. He also had diarrhea and had vomited several times before his admission. His previous health had been good, and he had no history of recent travel. Physical examination showed a temperature of 40.2°C and bronchial breath sounds at the right upper zone lung field. Chest x-ray confirmed right upper lobe consolidation (Figure, part A).

**Figure:**
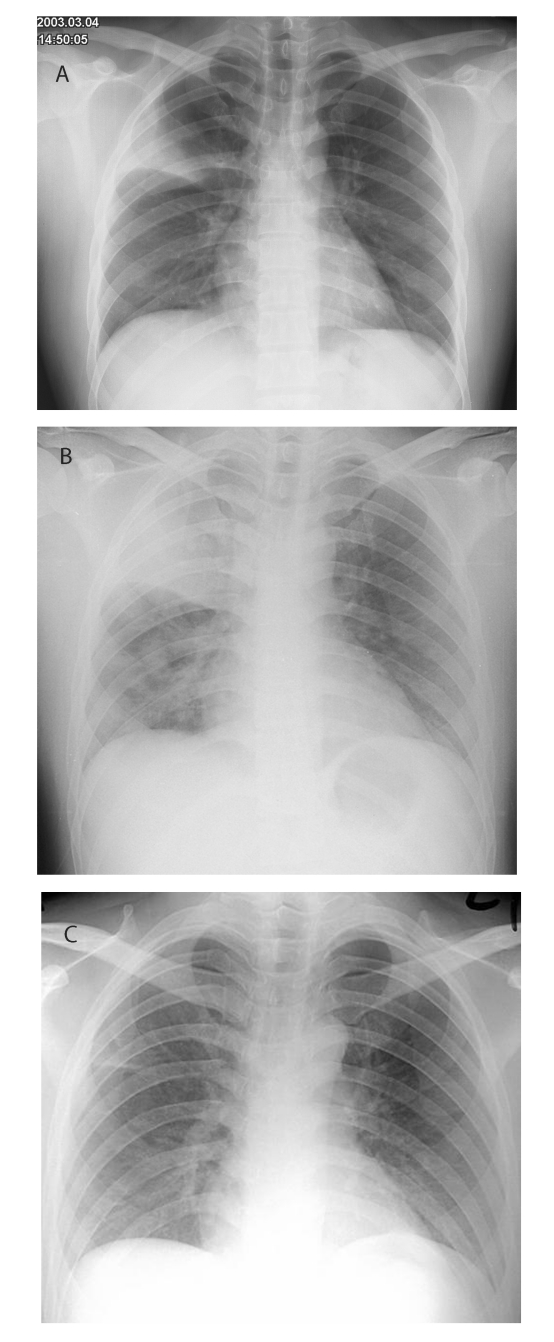
Chest radiographs performed A, at admission, B, on day 4, and C, on day 16 of hospitalization for index SARS case-patient, Prince of Wales Hospital.

A complete blood profile on admission showed a leukocyte count 3.1 x 10^9^/L, absolute neutrophil count 2.0 x 10^9^/L, lymphocyte count 0.7 x 10^9^/L, platelet count 112 x 10^9^/L, and hemoglobin 14.7 g/dL. The patient had mild renal impairment, with a creatinine of 119 μmol/L, urea and electrolytes within normal limits, and alanine transaminase mildly elevated at 90 IU/L (normal <58 IU/L). Bilirubin, alkaline phosphatase, and albumin levels were normal. C-reactive protein was 6.5 mg/L (normal <9.9 mg/L). A diagnosis of atypical or viral pneumonia was suspected because of the low leukocyte count and normal C-reactive protein. Other laboratory tests were performed, including blood, sputum, and urine cultures, nasopharyngeal aspirate for influenza and parainfluenza, indirect immunofluorescence for respiratory syncytial viral antigen detection, and atypical pneumonia titer (for adenovirus, psittacosis, Q fever, influenza A and B, and mycoplasma). The patient received treatment with intravenous amoxicillin-clavulanate and oral clarithromycin.

The patient was housed in a general medical ward with no specific isolation facility. After admission his high fever and productive cough, now with thick, yellowish sputum, persisted. He also complained of progressive dyspnea, headache, dizziness, generalized malaise, and myalgia. His pulse and blood pressure were normal, and his oxygen saturation was approximately 98% on room air. A sputum culture yielded normal oral flora, and sputum smears were negative for acid-fast bacilli. Nasopharyngeal aspiration was negative for influenza viruses A and B, respiratory syncytial virus, adenovirus, and parainfluenzavirus types 1, 2, and 3, with the use of commercial immunofluorescence assay. A chest radiograph on day 4 showed progression of pneumonia, with consolidation changes over the right upper and lower lobes (Figure, part B). A repeat complete blood profile showed a leukocyte count of 5.4 x 10^9^/L with persistent lymphopenia and a platelet count of 98 x 10^9^/L. Amoxicillin-clavulanate was therefore changed to intravenous cefotaxime, 1 g every 8 h; clarithromycin (500 mg twice a day) was continued. As the patient’s condition deteriorated progressively and he had difficulty in expectorating sputum, salbutamol, 0.5 g four times a day, driven by a jet nebulizer at 6 L of oxygen per min, was given to assist mucociliary clearance. His oxygen saturation remained normal without supplemental oxygen.

Starting from day 6, the patient’s fever and chest condition gradually improved. However, over the next 2 weeks, 138 persons (mostly healthcare workers) who had been in contact with him had onset of a similar illness with high fever and pneumonia. The patient was subsequently confirmed to be the index case-patient in this hospital outbreak of SARS (*1*). Three family members were also infected. Further history showed that he had visited a hotel in Kowloon, Hong Kong, where a 64-year-old physician from southern China had stayed for 2 days; this physician later died of severe atypical pneumonia 10 days after admission to a regional hospital in Kowloon (*2*). The cause of the illness was not known at the time of the physician’s death.

Our patient was identified as the index case-patient 5 days after the onset of this large outbreak at the Prince of Wales Hospital, as he was the first patient who had the characteristic clinical, radiologic, and laboratory features of SARS and had epidemiologic links with other infected persons. After 8 days, use of the nebulized bronchodilator was stopped because of the possibility of enhancing SARS transmission, and the patient was isolated in a private room with negative-pressure ventilation. Healthcare workers entering the room wore disposable gloves and N95 masks. After the patient completed a 7-day course of cefotaxime and a 10-day course of clarithromycin, his pneumonia recovered gradually, and serial chest radiographs confirmed resolution of his consolidation (Figure, part C). His diarrhea and other systemic symptoms also resolved spontaneously.

An immunofluorescence test for antibody against SARS-CoV subsequently confirmed an elevated titer of 1:5,120 in convalescent-phase serum collected on day 21 of illness. Polymerase chain reaction of nasopharyngeal aspirate was negative for coronavirus. Convalescent-phase serum was negative for other atypical pneumonia organisms, including adenovirus, psittacosis, Q fever, influenza A and B, and mycoplasma. Repeat complete blood count showed that lymphocytes and thrombocytes had returned to normal, along with serum creatinine and alanine transaminase levels.

The patient was isolated in a private room until day 27 of his hospital stay, when his nasopharyngeal aspirate and urine samples were confirmed to be negative for SARS-CoV. Repeat chest radiograph at follow-up 2 weeks later showed no residual parenchymal opacity, and the patient remained asymptomatic.

## Conclusions

This report describes the index patient responsible for the hospital outbreak in the Prince of Wales Hospital (*2*). He was linked to spread of the virus to more than 100 persons (*1*). This outbreak, together with similar events in Canada (*3*), Singapore (*4*) and other cities where the source of infection was also related to the Chinese physician (*5*), led to increased awareness of this emerging global infection caused by a novel coronavirus (*6*). The super-spread event in Prince of Wales Hospital caused by this patient was related to failure to apply isolation precautions, as the disease had not been recognized during the early part of his admission. The use of a nebulized bronchodilator may also have enhanced the spread of the virus in the ward, and this practice was stopped for patients with suspected SARS after this incident (*7*).

This case report illustrates the natural history of SARS in a young, previously healthy patient who received no specific therapy. His clinical features and laboratory parameters were similar to those of other patients with SARS (*2*–*5*). His clinical course followed a typical pattern with progression of pneumonia during the 2nd week of his illness (*8*). He was treated presumptively for bacterial community-acquired pneumonia with conventional antimicrobials (*9*), without antiviral agents or corticosteroids. He started to improve by the 3rd week and subsequently recovered uneventfully.

During the global outbreak in 2003, treatment of SARS was empiric. Several groups have reported the use of ribavirin (*2*–*5*,*7*,*8*) and corticosteroids (*2*,*3*,*7*,*8*,*10*,*11*) with generally favorable outcomes. Ribavirin has been associated with substantial adverse reactions, including hemolytic anemia, elevated transaminases, and bradycardia (*4*), and has demonstrated no in vitro activity against SARS-CoV (*12*). Further studies, preferably with a randomized, placebo-control design, are needed to address treatment of this disease, which has high attack rates and is frequently fatal.
